# Perceived effects of health status on sexual activity in women and men older than 50 years

**DOI:** 10.1186/1477-7525-12-43

**Published:** 2014-03-27

**Authors:** Gudrun Rohde, Kari Hansen Berg, Glenn Haugeberg

**Affiliations:** 1 Faculty of Health and Sport sciences, University of Agder, Servicebox 422, Kristiansand 4604, Norway; 2 Department of Rheumatology, Hospital of Southern Norway Trust, Kristiansand, Servicebox 416, Kristiansand 4604, Norway

**Keywords:** Health status, Sexual activity, Quality of life, Elderly

## Abstract

**Background:**

Sexual activity and enjoyment are considered to be important components of quality of life (QOL) for adults of all ages. However, limited data are available on the effects of health status on sexual activity in women and men older than 50 years. Thus, our aim was to explore the perceived effects of health status on sexual activity in women and men older than 50 years.

**Methods:**

For this purpose we used data from an age and gender matched control study initially designed to study QOL in patients with low-energy wrist fracture. We investigated patients with wrist fractures older than 50 years (n = 181), as well as age- and gender-matched controls (n = 226), who participated in the QOL study. There were minimal differences between patients and controls, thus the groups were pooled (mean age 67 years (8 SD)). Health-related quality of life (HRQOL) was assessed using SF-36 and 15D, and the global quality of life using the Quality of Life Scale (QOLS). To assess perceived effects of health status on sexual activity we used the question on sexuality from the 15D questionnaires. Group comparisons and logistic regression analyses were conducted.

**Results:**

The 15D question on sexuality was not answered by 25% of the participants. Health status having a large negative effect on sexual activity was reported by only 13% of the participants. In the multivariate analyses a large negative effect of health status on sexual activity was associated with higher age (60–69 years: OR = 5.7, 95% CI = 1.62–29.2; 70–79 years: OR = 3.60, 95% CI = 0.94–13.9; ≥80 years: OR = 9.04, 95% CI = 1.29–63.4), male gender (OR = 10.8, 95% CI = 3.01–38.9), weight (OR = 1.03, 95% CI = 1.00–1.07), low SF-36 PCS score (OR = 0.88, 95% CI = 0.37–0.93) and a low SF-36 MCS score (OR = 0.92, 95% CI = 0.88–0.96).

**Conclusion:**

Only a small proportion of the participants reported their health status to have a large negative effect on sexual activity. Furthermore, health status having a negative effect on sexual activity was associated with decreased HRQOL. Insights into this important topic may increase our awareness as health care workers and help us to address this aspect of QOL in this age group.

## Background

Quality of life (QOL) can be defined in various ways and may have different meanings or perspectives in studies. However, it is agreed that QOL is a subjective and multidimensional concept, which has physical, psychological, social and spiritual dimensions [1]. Sexual activity and enjoyment are considered to be components of these QOL dimensions, particularly the physical and psychological dimensions [1,2]. As people age, their physical and mental health decline, thus impaired health may also affect sexual activity both in women and men [3].

Previous studies in women and men older than 50 years have indicated that high QOL is associated with a higher level of sexual enjoyment [4] and sexual activity [5]. Regular sexual activity appears to be associated with younger age, higher income, being in a significant relationship and a lower body mass index (BMI), while satisfaction with sexual activity was associated with African-American race, low BMI and a higher mental score [6]. Furthermore, sexual problems seem to be associated with decreased QOL [7].

There is a lack of knowledge on the relationship between perceived health status and sexual activity, in particular in the population of women and men older than 50 years. Thus, our aim was to explore the effects of perceived health status on sexual activity in women and men in this age group.

## Methods

### Study population and data collection

The subjects were recruited from a prospective case–control QOL study of patients with low-energy wrist fractures and controls who were assessed at the osteoporosis center of a regional hospital in southern Norway. In this study a low-energy wrist fracture was defined as a fracture followed by minimal trauma falling from standing height or less. The patients were recruited in a two years period, 2004 and 2005, and were matched for age and gender with controls from the background population. The final study population comprised 181 wrist fracture patients (161 women and 20 men) and 226 controls (192 women and 34 men).

In the two year inclusion period, 324 patients with low-energy wrist fractures were treated at the hospital, and 249 of the patients were clinically examined at the Osteoporosis Centre. Among the 75 patients not examined at the osteoporosis centre, 14 patients were ineligible for bone mineral density (BMD) assessment because of poor mental or physical health, 13 patients were tourists, three patients were not invited for assessment for other reasons, and 45 patients declined to be assessed. Of the 249 patients examined at the Osteoporosis Centre, 181 met the inclusion criteria and were willing to enroll in this study. Of the 68 patients assessed at the osteoporosis centre but not included in the study, 17 were not able to self-report their health status because of dementia or confusion. Another two patients were tourists who did not reside in the geographic area, three patients were not invited to participate in the study for other reasons and 46 patients declined to participate, which give a response rate of 66%. The median time between fracture and examination at the osteoporosis centre for the 181 wrist fracture patients was 10 days. Controls were randomly identified in the national registry for the catchment area and were consecutively invited to participate in the study by mail [8-10]. The study protocol collected a broad spectrum of demographic and clinical data, which also included three QOL questionnaires: 15D, SF-36 and the Quality of Life Scale (QOLS) [8-11].

The patients were at inclusion asked to report the status of their demographic and clinical variables, and QOL, prior to their fractures. The controls were also asked about their status and habits at the time prior to inclusion. The data collected included demographic and clinical data, exercise levels (greater than 30 min exercise three times each week), smoking habits, co-morbidities (heart diseases, pulmonary diseases, neurological disorders, urogenital disorders, gastro-intestinal disorders, endocrine disorders, inflammatory joint disorders, connective tissue disorders, cancer, and mental disorders) and bone mineral density measure at femoral neck, total hip and lumbar spine (L2–L4) using dual-energy X-ray absorptiometry, as listed in Table 1. We also computed a summed score for co-morbidities to consider the number of diseases in each participant. The QOL data were collected as described below.

**Table 1:** Demographic, clinical, health status, health-related quality of life and global quality of life variables for all participants, i.e., respondents and non-respondents to item 15 on the 15D questionnaire

	**All (n = 407)**	**Respondents (n = 306)**	**Non-respondents (n = 101)**	**p-values**
**Demograhpic**				
Age (years)	67 (8)	65 (9)	73 (9)	<0.001
Age groups (years)				
50-59	121 (30%)	106 (35%)	15 (15%)	<0.001
60-69	131 (32%)	111 (36%)	20 (20%)	
70-79	122 (30%)	78 (25%)	44 (43%)	
80-	33 (8%)	11 (3%)	22 (22%)	
Women	353 (87%)	256 (84%)	97 (96%)	<0.001
Weight (kg)	72.0 (13.7)	73.3 (13.6)	68.3 (13.3)	0.001
BMI (kg/m2)	26.1 (4.3)	26.2 (4.3)	25.6 (4.4)	0.235
Menopause age (years)	49.3 (4.2)	49.2 (4.4)	49.5 (3.8)	0.522
Higher education (>13 years)	103 (25%)	90 (31%)	13 (13%)	0.001
Married/cohabiting	247 (61%)	215 (71%)	32 (32%)	<0.001
Regular exercise*	303 (75%)	237 (78%)	66 (66%)	0.022
Currently smoking	59 (15%)	48 (16%)	11 (11%)	0.231
**Co-morbidity**				
Mean total score for co-morbidity (range 0–6)	0.9 (0.9)	0.8 (0.9)	1.2 (1.1)	<0.001
**Clinical status**				
Osteoporoses**	97 (24%)	58 (19%)	39 (39%)	<0.001
≥1 fall in the previous year	142 (35%)	101 (39%)	41 (47%)	0.191
Previous fracture	194 (47%)	140 (46%)	54 (54%)	0.299
**Health status**				
Mean MHAQ***	1.05 (0.20)	1.04 (0.17)	1.09 (0.26)	0.015
**Health related quality of life**				
SF-36- PCS****	51 (9)	52 (9)	49 (10)	0.001
SF-36-MCS****	51 (9)	51 (9)	49 (11)	0.112
**Global quality of life**				
QOLS*****	95 (10)	96 (9)	93 (12)	0.042

*= more than 30 min three times each week.

**Osteoporosis at total hip and/or spine L2–L4.

***The MHAQ score ranges from 1 to 4 where 1 indicates a higher perception of the ability to perform daily living activities.

****The score for SF-36 ranges from 0 to 100 where 100 indicates a high HRQOL.

*****The QOLS score ranges from 16 to 112 where 112 indicates a high GQOL.

PCS = physical component summary, MCS = mental component summary, MHAQ = Modified Health Assessment Questionnaire, QOLS = quality of life scale.

Continuous variables are expressed as the mean and with standard deviation (SD) in brackets while categorical variables are expressed as numbers and percentage (%) in brackets. In the group comparisons, independent samples *t*-tests were used for continuous variables and chi-squared tests were used for categorical variables.

### QOL and health status measures

Item 15 in the 15D questionnaire was used to study the effect of health status on sexual activity [12]. The 15D questionnaire is a generic, multidimensional, standardized tool for evaluating health-related quality of life (HRQOL), which is used primarily as a single index measure but it can also be used as a profile utility measure. The 15D questionnaire captures the health status by assessing 15 dimensions: mobility, vision, hearing, breathing, sleeping, eating, speech, elimination, usual activities, mental function, discomfort and symptoms, depression, distress, vitality and sexual activity [2]. Each dimension is assessed by one question using five response categories. The questionnaire has been tested for psychometric properties in other studies, within several countries, including Norway [2,13].

Item 15 addresses the effects of health on sexual activity with the following response options.

My state of health:

1. … has no adverse effect on my sexual activity;

2. … has a slight effect on my sexual activity;

3. … has a considerable effect on my sexual activity;

4. … makes sexual activity almost impossible;

5. … makes sexual activity impossible.

To analyze the effects of health on sexual activity, we dichotomized the five responses to item 15 in the 15D instrument, which were related to sexual activity. Responses 1 and 2 were grouped into “no/little effects” and the other three categories were grouped into “large effects”.

HRQOL was also assessed using SF-36, which is a self-reported, generic questionnaire. SF-36 has eight domains: general health, bodily pain, physical functioning, role limitations (physical), mental health, vitality, social functioning and role limitations (emotional), which can be combined into a physical and mental sum scale that reflects physical and mental health. The physical component summary (PCS) and mental component summary (MCS) scales were used in this study. For incomplete questionnaires, substitution of missing values is based on the scale instructions given by the developers of the questionnaire [14,15]. The SF-36 scales were scored according to published scoring procedures and each was expressed as a value from 0 to 100 where 100 represented excellent health [14,15]. The questionnaire has been thoroughly tested for psychometric properties in other studies, within several countries, including Norway [14-16].

The global quality of life (GQOL) was assessed using QOLS, which is a 16-item, domain-specific instrument [17-19]. In QOLS, GQOL is understood as a broad range of human experiences related to one’s overall well-being and satisfaction [17,20]. The items are rated using a 7-point satisfaction scale. If 80% of the questions had been completed, the missing values in incomplete questionnaires were replaced with the mean value of answers given by respondents [21]. The questionnaire was scored by adding up the items to obtain a total score, which ranged from a minimum of 16 to a maximum of 112. Higher scores indicate better QOL [17]. The questionnaire has been tested for psychometric properties in other studies, within several countries including Norway [17,22].

The Modified Health Assessment Questionnaire (MHAQ) was used to measure a patient’s ability to perform daily living activities [23,24]. MHAQ comprises eight items that cover daily activities, including skills that demand a good arm function, e.g., dressing, lifting a full cup or glass to the mouth, and washing and drying the entire body. The total mean score range is 1–4 where 1 represents “without any difficulty” and 4 is “impossible” [23,24]. For incomplete questionnaires, the missing values were replaced with the mean value of the answered questions of the respondent when at least 6 out of 8 items had valid response, which is based on the scale instructions given by the developers of the questionnaire. A validated Norwegian version of the questionnaire was used [24,25].

### Statistical analysis

Statistical analyses were carried out using the Statistical Package for Social Sciences (SPSS) for Windows (version 19.0). Differences between two groups were analyzed using chi-squared tests for categorical variables and using *t*-tests for continuous variables. Independent samples *t*-tests were used to compare the differences in MHAQ scores, SF-36 scores and QOLS scores between respondents and non-respondents, and respondents reporting no/little effect versus large effect for item 15 in the 15D instrument.

Logistic regression analyses using the two comparison groups (respondents reporting large effect/little effect for item 15 in the 15D instrument) as the dependent variables were employed to identify significant associations (demographic, clinical and HRQOL variables; Table 1), which were retained for the final multivariate analysis of the effect of health status on sexual activity. Multivariate associations were explored by logistic regression analyses (backward procedure, clinically based), including the variables that were significant at a level of 0.15 in the bivariate analyses. The final model included the independent variables retained from the last step of the backward procedure in the logistic regression analyses. The R square was assessed by Nagelkerke R square. Associations were also tested for result consistency using enter and forward methods by logistic regression. The level of significance was set at p < 0.05.

### Ethics

The study was approved by the Regional Committee for Medical Research Ethics and by the National Data Inspectorate.

## Results

### Participants

Among all study participants, 101 (25%) did not answer the question on sexual activity (men 7%, women 28%, p = 0.001). There were no significant differences between wrist fracture patients and controls who answered the question on sexual activity, this tested for all (25% vs 24%, p = 0.80) and for men (5% vs 9%, p = 0.60) and women separately (28% vs 27%, p = 0.86). Furthermore, with regard to demographic and clinical differences between wrist fracture patients and controls there were only minor differences. Wrist fracture patients had more osteoporosis (p = 0.001), else there were no statistical significant differences between the two groups [9,10]. Thus, we present the results for patients and controls as a pooled group. The response rates for the other 14 questions in 15D ranged from 96.3% to 99.5%.

The socio-demographic, clinical and QOL characteristics of all participants, i.e., respondents and non-respondents to item 15 in the 15D questionnaire, are shown in Table 1. Non-respondents were characterized as follows: significantly older (p < 0.001), more likely women (p < 0.001), weighed less (p = 0.001), less highly educated (p = 0.001), more likely to live alone (p = 0.001), exercised less (p = 0.022), more osteoporosis(p<0.001) and suffered more from other diseases (p < 0.001), and reported higher MHAQ (p = 0.015), lower SF-36 PCS (p = 0.001) and lower QOLS (p = 0.042).

### Health status and sexual activity

**Figure 1:**
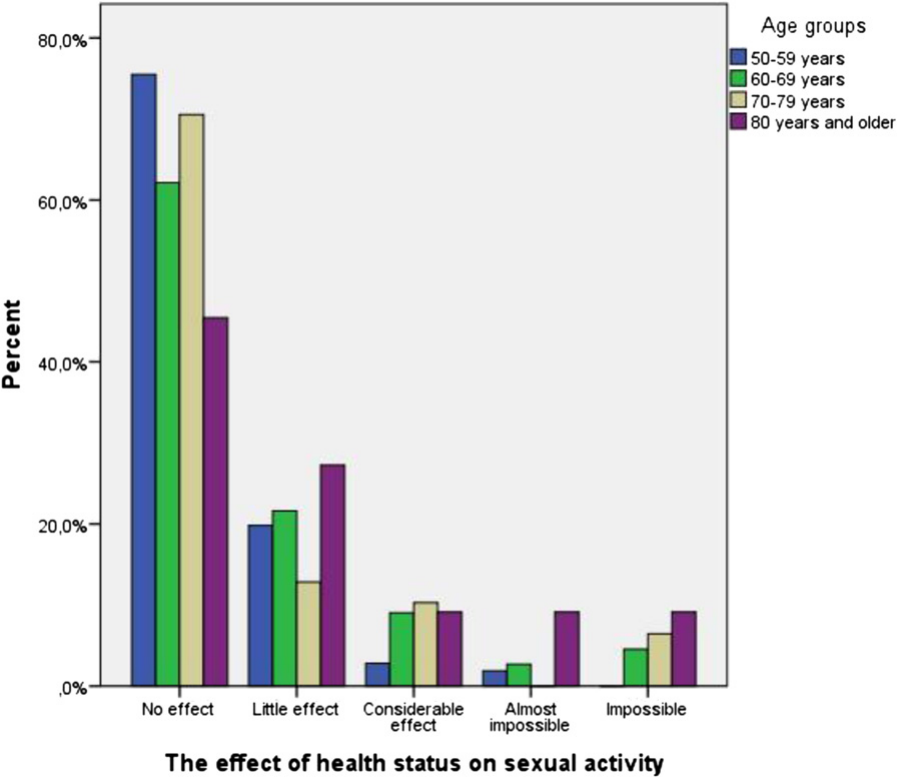
The effect of health status on sexual activity (item 15 in the 15D questionnaire), stratified for age-groups.

Of the 306 respondents to item 15 in the 15D questionnaire, no effect on sexual activity was reported by 68% (wrist fracture patients 76% and controls 62%), little effect by 19% (wrist patients 14% and controls 23%), considerable effect by 7% (wrist patients 5% and controls 9%), while 2% (wrist patients 2% and controls 2%) reported that sexual activity was almost impossible and 4% (wrist patients 3% and controls 4%) reported that sexual activity was impossible. The chi-squared test detected no significant difference between the wrist fracture patients and controls (data not shown). Figure 1 shows the results for the age-groups (p = 0.058).

### No\little versus large negative effect on sexual activity

The socio-demographic, clinical and QOL characteristics of respondents who reported no/little effect of health status on sexual activity versus respondents who reported large effect are described in Table 2. Respondents who reported that their health status had a large effect compared with respondents who reported no/little effect on sexual activity were: older (p = 0.004), more likely to be men (p *=* 0.032), weighed more (p = 0.007), had a higher BMI (p = 0.018) and exercised less (p = 0.001); they also reported lower SF-36 PCS (p < 0.001), lower SF-36 MCS (p = 0.001) and lower QOLS (p < 0.001).

**Table 2:** Demographic, clinical, health status, health-related quality of life and global quality of life variables for all participants who reported that their health status had no/little effect and a large effect on their sexual activity

	**No\little effect (n = 267)**	**Large effect (n = 39)**	p-values***
**Demograhpic**			
Age (years)	64 (9)	69 (8)	0.004
Age groups (years)			
50-59	101 (38%)	5 (13%)	0.015
60-69	93 (35%)	18 (46%)	
70-79	65 (24%)	13 (33%)	
80-	8 (3%)	3 (8%)	
Women	228 (85%)	28 (72%)	0.032
Weight (kg)	72.5 (13.0)	78.7 (16.3)	0.007
BMI (kg/m2)	26.0 (4.1)	27.8 (5.1)	0.018
Menopause (years)	49.2 (4.5)	49.6 (3.9)	0.658
Higher education (>13 years)	81 (32%)	9 (24%)	0.375
Married/cohabiting	186 (70%)	29 (74%)	0.593
Regular exercise*	215 (81%)	22 (56%)	0.001
Currently smoking	43 (16%)	5 (13%)	0.592
**Co-morbidity**			
Mean total score for co-morbidity (range 0–6)	0.8 (0.9)	1.1 (0.9)	0.062
**Clinical status**			
Osteoporois**	49 (18%)	9 (23%)	0.489
≥1 fall in the previous year	89 (39%)	12 (39%)	0.958
Previous fracture	122 (46%)	18 (46%)	0.545
**Health status**			
Mean MHAQ***	1.0 (0.2)	1.1 (0.2)	0.567
**Health related quality of life**			
SF-36 - PCS****	53 (8)	44 (9)	<0.001
SF-36 – MCS****	52 (8)	44 (12)	0.001
**Global quality of life**			
QOLS*****	97 (9)	89 (11)	<0.001

*= more than 30 min three times each week.

**Osteoporosis at total hip and/or spine L2–L4.

***The MHAQ score ranges from 1 to 4 where 1 indicates a higher perception of the ability to perform daily living activities.

****The score for SF-36 ranges from 0 to 100 where 100 indicates a high HRQOL.

*****The QOLS score ranges from 16 to 112 where 112 indicates a high GQOL.

PCS = physical component summary, MCS = mental component summary, MHAQ = Modified Health Assessment Questionnaire, QOLS = quality of life scale.

Continuous variables are expressed as the mean and with standard deviation in brackets (SD) while categorical variables are expressed as numbers and percentage (%) in brackets. In the group comparisons, independent samples *t*-tests were used for continuous variables and chi-squared tests were used for categorical variables.

In the multivariate analyses (backward method), a large negative effect of health status on sexual activity was associated with higher age (60–69 years: OR = 5.7, 95% CI = 1.62–29.2; 70–79 years: OR = 3.60, 95% CI = 0.94–13.9; ≥80 years: OR = 9.04, 95% CI = 1.29–63.4), male gender (OR = 10.8, 95% CI = 3.01–38.9), weight (OR = 1.03, 95% CI = 1.00–1.07), low SF-36 PCS score (OR = 0.88, 95% CI = 0.37–0.93) and a low SF-36 MCS score (OR = 0.92, 95% CI = 0.88–0.96) (Table 3). The same patterns were obtained using enter and forward methods in the multiple logistic regression analyses.

**Table 3:** Multivariate logistic regression model showing the adjusted associations between demographic, clinical and quality of life variables and reporting that health status had a large effect on sexual activity in the 15D question

	**Full model or (95% CI)**	**p-value**	**Final model or (95% CI)**	**p-value**
Age groups (years)				
50–59 ref-		0.031		0.037
60–69	6.78 (1.83–25.1)	0.004	5.70 (1.62–29.2)	0.007
70–79	4.33 (1.07–17.6)	0.040	3.60 (0.94–13.9)	0.062
≥80	8.10 (0.89–74.0)	0.064	9.04 (1.29–63.4)	0.027
Men	4.78 (1.43–16.0)	0.001	10.8 (3.01–38.9)	<0.001
Weight (kg)	1.04 (1.00–1.08)	0.028	1.03 (1.00–1.07)	0.041
Higher education (>13 years)	1.61 (0.54–4.81)	0.392		
Married/cohabiting	1.69 (0.55–5.27)	0.363		
Regular exercise*	1.75 (0.63–4.87)	0.283		
Mean total score for co-morbidity (range 0–6)	0.58 (0.33–1.03)	0.061		
Wrist fracture group	0.76 (0.27–2.11)	0.597		
SF-36 PCS**	0.89 (0.83–0.94)	<0.001	0.88 (0.37–0.93)	<0.001
SF-36 MCS**	0.93 (0.88–0.98)	0.007	0.92 (0.88–0.96)	0.001
QOLS***	0.98 (0.92–1.04)	0.506		
R^2^	44.5%		41.0%	

*= exercise for more than 30 min three times each week.

**The score for SF-36 ranges from 0 to 100 where 100 indicates a high HRQOL.

***The QOLS score ranges from 16 to 112 where 112 indicates a high GQOL.

PCS = physical component summary, MCS = mental component summary, QOLS = quality of life scale.

The full model included all of the selected variables entered into the model while the final model included variables in the final step using the backward procedure.

## Discussion

The main findings of this QOL study of a relatively healthy population of women and men older than 50 years (mean age 67 years) were that only a small proportion (13%) reported that their health status had a large negative effect on sexual activity. A large negative effect on sexual activity was associated with being older, male gender, increased weight and lower physical and mental health.

Of those who answered the question on sexual activity, the majority reported that their health status had no/little effect on their sexual activity. It is most likely that the participants in our study included a relatively healthy group of women and men older than 50 years and our findings are influenced by that [10,11]. In a Finnish national health survey (n = 6681), 12.5% of subjects reported sexual problems [26], which is in line with our findings. This contrasts with the study by Helland et al. [12], which found that as many as 31% of a rheumatoid arthritis (RA) cohort (21% non-respondents) reported that their health status had a significant negative impact on sexual activity [12]. This highlights the severe effects of RA on HRQOL and sexual activity. In our study increased co-morbidity did not show a significant association with health status having a large negative effect on sexual activity (p = 0.06). Previous studies have indicated that co-morbidities such as cardio-vascular disorders, diabetes, ankylosing spondylitis (AS) and mental health problems are correlated with sexual problems and dysfunctions [27,28].

Previous studies have reported sexual problems to increase with age [3], which partly agrees with our study. As shown in Figure 1 there was a greater tendency for the oldest age-group of subjects to report that health status had a negative effect on sexual activity compared with the younger one. It is important to note that sexual activity remains a significant part of relationships also in later life [3]. In our study, increased weight was independently associated with health status having a large negative effect on sexual activity. This finding agrees with Addis et al. who reported a significant association between low BMI and regular sexual activity, and low BMI and satisfaction with sexual activity [6], which was also reported by Heiman [27].

In our study, reduced physical and mental HRQOL were independently associated with health status having a negative effect on sexual activity, whereas there was no significant association with GQOL. This might be attributed to the phrasing of the sexual activity item, which focuses on health status rather than the GQOL or overall QOL [15,17,20]. These associations between HRQOL and sexual activity have also been reported in other studies [3,7,29].

The low number of patients reporting their health status to have a “large negative effects on sexual activity” may have weakened the multivariable regression model. The consequence of this is broad and overlapping confidence intervals e.g. in the age groups. This limitation should therefore be taken into account when interpreting our results.

It may be surprising that as many as 25% of the subjects did not answer the question on sexual activity, although they participated in a QOL study. This may partly be explained by that these subjects did not consider this question to be relevant to their life, or that the participants found this question on their sexual activity to be too sensitive. This view is supported by that the response rate for the other 14 questions in 15D ranged from 96.3% to 99.5%. The relatively low response rate on sexuality has also been reported by others. In the study of RA patients with mean age of 58.5 years and disease duration of 13.4 years, Helland et al. [12] found that 20% of the patients did not respond to the question on sexuality in the 15D questionnaire, while Healy at al [28] in their study found that 10% of their AS patients did not complete the questions about their sexual relationship. Finding a suitable partner, age and the relationship status have been reported to have significant effects on having regular sexual activity [7]. Addis et al. [6] reported that younger age and having a significant relationship were associated with having regular sexual activity. The gender differences were also striking in our study. We found that significantly more men (92%) than women (73%) answered the question on sexual activity. The findings might reflect the fact that females tend to outlive their partners, and hence lack of applicability may be the main reason that women did not responded to the question [28]. This has also been observed by other studies [12].

There is a methodological limitation in our study as fracture patients at inclusion were asked to report their health condition prior to fracture. This is important as retrospective evaluations can be biased by recall problems and response shifts due to the fracture [9-11]. This may have influenced our results. However because the data were collected shortly after the fracture, in median 10 days, we believe this may have had limited impact on the results. The minor differences between fracture patients and the controls support this view.

The strengths of this study were that all the participants were consecutively recruited from the same geographical area and the numbers of participants were relatively high. The pooling of wrist fracture patients and controls is justified as there were only minor differences between wrist fracture patients and the matched controls [8-10].

A major limitation of our study was the cross-sectional study design, which does not permit any causal interpretations and can only establish associations between dependent and independent variables. Furthermore, the study was female dominant due to a higher prevalence of wrist fractures among women. We recruited the participants consecutively and none were excluded due to gender. The female dominance might limit the ability to generalize the findings to all subjects aged 50 years and older. A third limitation is that the effect of health status on sexual activity was captured by only one survey item. Sexual activity and enjoyment are complex phenomena, which should ideally be measured using several items to capture various aspects [3,6,29]. And fourth, the participants were relatively healthy women and men older than 50 years. The exclusion criteria applied in this study meant that the non-participants were older and a more nuanced picture may have emerged if these patients had been included in the study. And at last, the participants were recruited to a study of QOL in fracture patients and the aim of the present study is one out of several\more focuses.

## Conclusion

In our study only a minority of the participants reported that their health status had a large negative effect on their sexual activity. A large negative effect on sexual activity was associated with being older, male gender, increased weight and lower physical and mental health, and it seems to be associated with HRQOL. Further studies are required to elucidate sexual activity as a component of QOL, especially in elderly individuals.

## Abbreviations

AS: Ankylosing spondylitis; BMD: Bone mineral density; BMI: Body mass index; GQOL: Global quality of life; HRQOL: Health-related quality of life; MCS: Mental component summary; PCS: Physical component summary; RA: Rheumatoid arthritis; SF-36: Short form-36; s-score: Standard difference score; SPSS: Statistical package for social sciences; QOLS: Quality of life scale.

## Competing interests

The authors declare that they have no competing interests.

## Authors’ contributions

GR analyzed the data and wrote the manuscript. GH was the principal investigator for the study. GH and KHB contributed to the content of the paper. All authors critiqued revisions of the paper and approved the final manuscript.
